# Recurrent Nasopharyngeal Chordoma Stabilized With Afatinib: A Case Report

**DOI:** 10.7759/cureus.96909

**Published:** 2025-11-15

**Authors:** Christopher C Chen, Vincent Yeung

**Affiliations:** 1 Internal Medicine, Rutgers New Jersey Medical School, Newark, USA; 2 Hematology and Oncology, Rutgers New Jersey Medical School, Newark, USA

**Keywords:** afatinib, brachyury, chordoma, epidermal growth factor receptor (egfr), tyrosine kinase inhibitor (tki)

## Abstract

Chordomas are rare malignant tumors originating from notochordal remnants that give rise to midline structures of the axial skeleton, including the skull base, mobile spine, and sacrococcyx. Extraosseous soft tissue chordomas near the skull base are extremely rare. We present a case of a 58-year-old Hispanic male with a two-month history of dysphagia, mild odynophagia, difficulty breathing, voice changes, decreased appetite, and weight loss. Imaging revealed a 3.9 cm heterogeneously enhancing retropharyngeal mass. He underwent transoral resection, and pathology confirmed chordoma. He remained disease-free postoperatively until surveillance imaging found recurrence. Over nearly 10 years, the patient underwent three additional surgical excisions for recurrent disease and received proton-beam radiation therapy. He was treated with imatinib, which was stopped due to intolerable adverse effects, followed by dasatinib, which failed to prevent disease progression. He was ultimately switched to afatinib and has since demonstrated relatively stable disease for two years, highlighting the potential of this epidermal growth factor receptor (EGFR) inhibitor as a promising targeted therapy in the context of limited systemic treatment options for chordomas.

## Introduction

Chordomas are rare malignant tumors of embryonic notochordal origin that arise from the axial skeleton in more than 90% of cases. They typically involve the base of the skull, vertebral bodies, and sacrococcyx in roughly equal distribution [1]. Vertebral body and sacrococcygeal chordomas exhibit a 2:1 male predominance, while skull base chordomas do not follow such a pattern [1]. Epidemiologically, chordomas occur at a rate of approximately 0.8 cases per million individuals per year, predominantly affecting patients between 40 and 60 years of age. The median age at presentation is 59 years, with five‑year survival rates ranging from 50% to 85% depending on lesion location [1-3]. Although most cases occur within this age range, fewer than 5% of chordomas are reported in children and adolescents, who tend to have a poorer prognosis due to the predominance of poorly differentiated chordomas [3,4]. Chordomas in older individuals tend to be of the conventional type [3,4]. Overall survival has been estimated at 67.6% at five years, 39.9% at 10 years, and 13.1% at 20 years [5]. The current World Health Organization (WHO) classification system defines the following three main types of chordomas: conventional, dedifferentiated, and poorly differentiated [3]. Each subtype is characterized by high local recurrence and metastasis rates, with non-cranial chordomas exhibiting a metastatic rate approaching 40% [1].

Treatment for chordomas has historically focused on radical surgical resection with wide margins while preserving essential anatomy, often requiring a multidisciplinary team consisting of neurosurgeons and other specialists. Despite advances in radiotherapy and chemotherapeutics, surgical intervention remains the gold standard, and the quality of resection is the most critical prognostic factor [6]. In recent years, advancements in the understanding of the molecular pathways of chordomas have paved the way for the adoption of several targeted therapies, focusing on receptor tyrosine kinases such as epidermal growth factor receptor (EGFR), platelet-derived growth factor receptor (PDGFR), and brachyury, a transcription factor almost exclusively expressed in chordomas [6]. This case highlights the use of afatinib, an EGFR inhibitor with potential dual activity against brachyury.

## Case presentation

**Figure 1:**
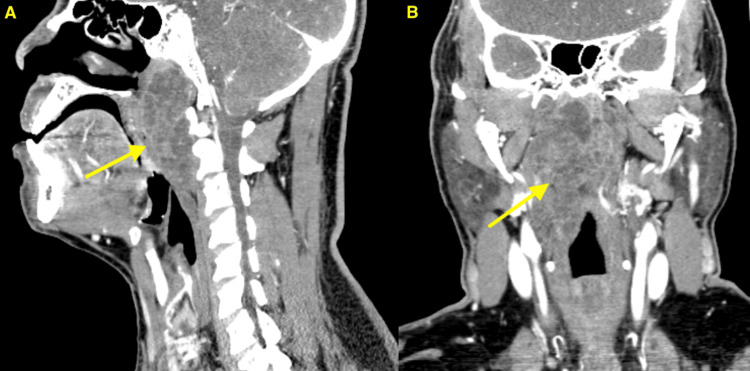
CT findings of the mass. (a) Sagittal view and (b) coronal view show a large heterogeneously enhancing soft tissue mass (yellow arrows) extending from the central and right aspect of the nasopharynx with contiguous inferior extension into the oropharyngeal soft tissues terminating just inferior to the right palatine tonsillar pillar.

A 58-year-old Hispanic male with a past medical history of type II diabetes and hypertension initially presented to our institution in 2015 with a two-month history of progressive dysphagia to solid foods, mild odynophagia, and difficulty breathing. Associated symptoms included voice changes, decreased appetite, and weight loss. His family history was significant only for diabetes, and his past surgical history was unremarkable. Social history was notable for a 10-pack-year cigarette smoking history. Physical examination, including flexible fiberoptic laryngoscopy, revealed a large, right-sided oropharyngeal submucosal mass measuring 5 cm. Computed tomography (CT) of the neck identified a 3.5×2.4×3.9 cm heterogeneously enhancing retropharyngeal mass extending from the nasopharynx to the oropharynx (Figures 1A, 1B). An initial fine-needle aspiration (FNA) biopsy of the lesion was negative for malignancy. The patient subsequently underwent transoral resection of the retropharyngeal mass. Surgical pathology demonstrated epithelioid cells in a myxoid/chondroid-type matrix. Immunohistochemical staining was positive for S-100, p63, calponin, cytokeratin anti-epithelial antibody cocktail 1/3 (AE1/3), epithelial membrane antigen (EMA), and brachyury, supporting the diagnosis of chordoma.

The patient had an uneventful postoperative recovery, with radiological evidence of gross total resection of the mass, until one year later, when he began experiencing increased nasal obstruction that affected his sleep. CT again demonstrated a heterogeneously enhancing mass in the nasopharyngeal and oropharyngeal soft tissues, for which the patient underwent another resection. Surgical pathology was again positive for chordoma. Due to the recurrent nature of his disease, the patient underwent proton-beam radiation therapy (RT), receiving a total dose of 72 Gy.

Two years later, the patient developed a slowly enlarging 2 cm mobile nodule below the right neck incision site, which was excised and confirmed to be chordoma. Surveillance imaging at the three-year mark re-demonstrated a recurrent chordoma in the right parapharyngeal space that was resected. Several months later, the patient reported recurring bilateral nasal congestion and a new right neck mass and underwent his third full surgical resection following imaging confirmation of recurrence.

The patient returned with worsening dysphagia and headaches and underwent his fourth resection of the recurrent chordoma two years following the prior resection. The postoperative course was complicated by a right lower lobe subsegmental pulmonary embolism and bilateral transverse sinus thrombosis, which were treated with heparin followed by apixaban. Tissue-based next-generation sequencing (NGS) was performed to evaluate for actionable mutations and returned only a cyclin-dependent kinase inhibitor 2A and 2B (CDKN2A/B) mutation, which is associated with cell cycle dysregulation but currently lacks a targeted therapeutic option in chordoma. Given his repeated recurrences and residual tumor burden, the patient was started on imatinib at 400 mg twice daily, which was shortly discontinued after one month due to adverse effects, including right facial swelling and grade three arthralgias, despite a dose reduction to 400 mg/day.

The chemotherapeutic regimen was switched to dasatinib at 70 mg twice daily, which again required a dose reduction to 70 mg/day due to right neck swelling and myalgias after three weeks. A surveillance magnetic resonance imaging (MRI) scan two months later revealed a local recurrence of the chordoma, with extension across the midline into the left parapharyngeal space. Both neurosurgery and otolaryngology deemed the patient not a surgical candidate for further resection. The patient was eventually switched to the EGFR tyrosine kinase inhibitor afatinib at 40 mg/day, which he tolerated well aside from grade one diarrhea and grade two acneiform rash. Clinically, he improved with reduced neck pain and a smaller right neck lesion on examination. The patient reported improved symptoms with only mild side effects of diarrhea and continued afatinib at 30 mg/day. He has enjoyed a largely stable disease course following initiation of afatinib, requiring a brief course of RT for oligo-progressive disease in mediastinal nodes in early 2025. A summary of the patient's clinical course, presented in chronological order, is provided in Table 1.

**Table 1:** Summary of the patient’s clinical course (2014-2025). RT: radiation therapy

Date	Event	Outcome
Nov 2014	CT neck: 3.5×2.4×3.9 cm prevertebral/oropharyngeal mass	Initial identification of lesion
Jan 2015	Presentation with progressive dysphagia, odynophagia, dyspnea	Clinical evaluation initiated
Mar 2015	Transoral resection of retropharyngeal mass	Pathology confirmed chordoma; gross total resection
Jun 2016	CT neck: recurrent mass	Local recurrence
Aug 2016	Endoscopic transnasal-transoral-transcervical resection	Recurrent chordoma resected
Oct-Dec 2016	Proton-beam RT	Completed; severe mucositis
Jul 2017-Mar 2018	Surveillance MRI	Stable postoperative enhancement
Sep 2018	Excision of right-neck nodule	Recurrent chordoma; no residual disease
Aug 2020	MRI: bilateral retropharyngeal lesions	Suggestive of recurrence
Jul 2021	Endoscopic resection (right parapharyngeal + adjacent sites)	Recurrent chordoma; temporary relief
Apr 2022	Endoscopic transoral/transcervical resection	Positive margins; partial resection
Dec 2022	CT: carotid encasement, necrotic nodes, lung nodules	Progressive disease
Mar 2023	Extended far-lateral skull-base resection + right neck dissection (II-IV)	Postoperative pulmonary embolism + dural sinus thromboses; anticoagulated
Apr 2023	Imatinib 400 mg twice daily started	Stopped after one week for toxicity
May 2023	Imatinib 400 mg daily re-challenge	Discontinued after two weeks; intolerance
Jul 2023	Dasatinib 70 mg twice daily → 70 mg daily	Myalgias; progression on therapy
Sep 2023	Afatinib 40 mg daily initiated	Clinical improvement; mild diarrhea and rash
Nov 2023	Afatinib dose reduced to 30 mg daily for mild transaminitis	Transaminases normalized; continued therapy
Dec 2023	MRI: mixed response	Continued afatinib
Apr 2024	Brief afatinib hold → resumed	Stable disease
Jan 2025	Proton RT to oligo-progressive disease	Well tolerated
Sep 2025	Latest follow-up	Stable disease on afatinib; minimal toxicity

## Discussion

Complete surgical resection with clear margins is widely regarded as the mainstay of chordoma treatment. However, this remains challenging due to the malignancy’s propensity for local recurrence and metastasis, even after repeated resections, as seen in our patient. Surgical debulking procedures are typically performed with the intent of removing as much tissue as possible while preserving vital structures, often at the cost of significant morbidity. These surgeries left our patient with vocal cord paralysis and percutaneous endoscopic gastrostomy (PEG) tube dependence. The debilitating nature of surgical intervention can profoundly impact a patient's quality of life, though it remains the first-line standard of care treatment for most chordoma patients. More than 50% of patients have at least local recurrence of the disease after surgery [7]. In these advanced settings, such as in our patient with recurrent disease despite repeated surgeries and RT, systemic therapy may be utilized, although data remain limited.

Many systemic treatments have been studied in clinical settings, though cytotoxic chemotherapies are not generally recommended for most chordoma patients, except for those with the aggressive, dedifferentiated subtype [8]. The current literature discusses numerous molecular targets, such as brachyury, EGFR, platelet-derived growth factor receptor (PDGFR), phosphatidylinositol 3-kinase/mammalian target of rapamycin (PI3K/mTOR), mitogen-activated protein kinase (MAPK), mesenchymal-epithelial transition factor (MET), and others [8].

Imatinib was the first molecular therapy to demonstrate some activity in chordomas. A phase II study in 2006 evaluated imatinib 800 mg/day in 50 patients with advanced chordoma, finding that, at the six-month mark, one patient had a partial response, while 35 patients had stable disease; median progression-free survival (PFS) was nine months and median overall survival (OS) was 35 months [7-9]. Unfortunately, our patient could not tolerate the side effects of imatinib, even after a dose reduction to 400 mg/day. A retrospective case series confirmed the activity of imatinib in PDGF-positive chordoma, with 21% of patients remaining progression-free at 18+ months [7].

Dasatinib, another tyrosine kinase inhibitor, was studied in 32 patients in a 2016 phase II trial, which demonstrated a PFS rate of 54% at six months and a five-year OS rate of 18% [10,11]. After two months of dasatinib, our patient experienced rapid progression involving the contralateral nasopharynx.

The patient was switched to the EGFR tyrosine kinase inhibitor afatinib, which has demonstrated activity in chordomas. Unlike some other EGFR inhibitors, afatinib contains a unique electrophilic group that promotes complete and sustained inhibition of receptor phosphorylation [12]. Evidence suggests afatinib may be the most effective EGFR inhibitor due to its concurrent breakdown of brachyury, enhancing its anti-tumor effect [10,12]. Brachyury is a transcription factor encoded by the T gene necessary for notochord development. After embryogenesis, brachyury is typically silenced, and its reactivation in chordomas serves as a highly specific tumor marker [6]. A study by Hsu et al. established the first xenograft model of sacral chordoma in a mouse, identifying brachyury as a critical component of chordomagenesis; silencing it led to complete senescence in in vitro chordoma cell lines [13]. Higher brachyury expression has also been associated with shorter PFS (five months vs. 13 months) [14].

Vaccines targeting brachyury, such as the yeast-based vaccine GI-6301, showed promising results in a phase I trial but did not improve overall response in a phase II trial [15]. Other experiments, such as the TAEK-VAC-HerBy vaccine, are currently underway to study the efficacy of such vaccines. A multicenter phase II trial involving 47 patients with EGFR-expressing chordoma from 2018 to 2022 showed promising results using afatinib as either first-line or later-line tyrosine kinase inhibitor as follows: PFS was 40% at 12 months in the first-line cohort and 38.5% at nine months in the later-line cohort [16]. Our patient began afatinib after failing both imatinib and dasatinib and has since enjoyed stable disease for more than two years, surpassing the median PFS reported in existing studies. In addition to radiographic stability, he also reported meaningful symptomatic improvement, including reduced neck pain and better functional status. These patient-reported outcomes are particularly important in chordoma treatment, where the preservation of quality of life remains a key therapeutic goal.

Afatinib may exert its therapeutic effects through "off-target" mechanisms, including the downstream suppression of signaling pathways that intersect with brachyury-mediated transcriptional activity. In vivo studies have demonstrated that afatinib can induce degradation of brachyury, inhibit cellular proliferation, and promote cell cycle arrest in chordoma models [12]. This dual targeting of EGFR and brachyury suggests a potentially synergistic mechanism of action that may be beneficial in cases resistant to standard therapies. Notably, the only actionable mutation identified in our patient was a deletion in the tumor suppressor genes cyclin-dependent kinase inhibitor 2A and 2B (CDKN2A/B). These genes regulate cell-cycle arrest, and their loss is associated with increased proliferation and poor prognosis in multiple malignancies. While there are limited data linking CDKN2A/B loss to chordomagenesis, studies in other solid tumors, such as glioma, have suggested that CDKN2A/B deletion may confer resistance to certain therapies. In fact, CDKN2A deletion is part of the mechanism that contributes to EGFR inhibitor resistance [17]. Whether CDKN2A/B mutations interact with EGFR or brachyury-driven oncogenesis remains unclear but warrants further investigation, especially in patients like ours who respond to therapies despite lacking canonical mutations.

## Conclusions

Standardized medical therapy to treat chordomas has not yet been established due to the scarcity of patient data and the infeasibility of large-scale randomized controlled trials. Our case, one of the few reported instances of nasopharyngeal chordoma stabilized on afatinib, highlights this EGFR inhibitor as a promising targeted therapy in a patient whose disease progressed after repeated surgical resections, radiotherapy, and multiple tyrosine kinase inhibitors. Afatinib’s observed clinical benefit in our patient, despite the absence of classical EGFR mutations, underscores the need for further study and continued documentation of real-world responses to these emerging agents. Prospective studies and collaborative registry-based data collection efforts will be critical to improving evidence-based management of rare tumors like chordoma.
